# “Macrobot”: An Automated Segmentation-Based System for Powdery Mildew Disease Quantification

**DOI:** 10.34133/2020/5839856

**Published:** 2020-11-05

**Authors:** Stefanie Lück, Marc Strickert, Maximilian Lorbeer, Friedrich Melchert, Andreas Backhaus, David Kilias, Udo Seiffert, Dimitar Douchkov

**Affiliations:** ^1^Leibniz Institute of Plant Genetics and Crop Plant Research (IPK), Correnstr. 3, 06466 Seeland, Germany; ^2^Physics Institute II, University of Giessen, Heinrich-Buff-Ring 16, 35392 Giessen, Germany; ^3^Julius Kühn Institute for National and International Plant Health, Messeweg 11/12, 38104 Braunschweig, Germany; ^4^Fraunhofer Institute for Factory Operation and Automation (IFF), Sandtorstr. 22, 39106 Magdeburg, Germany

## Abstract

Managing plant diseases is increasingly difficult due to reasons such as intensifying the field production, climatic change-driven expansion of pests, redraw and loss of effectiveness of pesticides, rapid breakdown of the disease resistance in the field, and other factors. The substantial progress in genomics of both plants and pathogens, achieved in the last decades, has the potential to counteract this negative trend, however, only when the genomic data is supported by relevant phenotypic data that allows linking the genomic information to specific traits. We have developed a set of methods and equipment and combined them into a “Macrophenomics facility.” The pipeline has been optimized for the quantification of powdery mildew infection symptoms on wheat and barley, but it can be adapted to other diseases and host plants. The Macrophenomics pipeline scores the visible powdery mildew disease symptoms, typically 5-7 days after inoculation (dai), in a highly automated manner. The system can precisely and reproducibly quantify the percentage of the infected leaf area with a theoretical throughput of up to 10000 individual samples per day, making it appropriate for phenotyping of large germplasm collections and crossing populations.

## 1. Introduction

Cereals, which include wheat, barley, rice, maize, rye, oats, sorghum, and millet, have been the primary component of humans' diet delivering more than 50% of the world's daily caloric intake [1]. Like any other plant, these species are under constant attack by a vast number of pathogens. However, because the impact of cereal diseases is proportional to the importance of these crops for human nutrition, they are of exceptional interest to plant pathologists and breeders.

Precise and sensitive phenotyping is one of the critical requirements for modern breeding and functional genomics studies. Many of the desired traits are polygenic by nature, and their manifestation depends on the cumulative effect of several factors with a small to moderate effect. The quantitative disease resistance of the plants against pathogens is a typical example of a complex polygenic trait. Although this type of resistance is usually less efficient than the strong R-gene-based resistance, it is nevertheless a desired trait because of its durability on the field, and in high contrast to the R-gene resistance, it is effective against all races of a particular pathogen and even against different pathogen species. However, studying the underlying mechanisms of the quantitative resistance is seriously challenged by the complexity of this phenomenon [2, 3]. The accessibility of the genomic information for several host and pathogen species greatly facilitates these studies but, on the other hand, introduced an enormous amount of data that needs to be tested and functionally validated. Thus, the ability of high throughput becomes an essential requirement for the new systematic phenotyping, and the term “phenomics” was coined to describe this approach.

The natural disease resistance is, besides the high yield and abiotic stress resistance, one of the most desired crop traits since the beginning of the agriculture. The breeders invested significant efforts in improving these traits, and as a result, the modern crop cultivars are usually outperforming their wild progenitors in nearly all aspects. However, unlike other factors that may influence plant performance, the pathogens actively develop and modify strategies to evade the host defense mechanisms in a process sometimes called “evolutionary arms race”.

Powdery mildew (PM) is a disease caused by a diverse group of obligate biotrophic fungi that lead to extensive damage to various crop plants, including cereals. *Blumeria graminis* is the causative agent of the powdery mildew disease of wheat and barley [4]. Like most of the obligate biotrophs, *B. graminis* shows extreme host specificity. The so-called *formae speciales* (f.sp.) have specialized virulence for particular plant species, e.g., for barley (*B. graminis* f.sp. *hordei*) or wheat (*B. graminis* f.sp. *tritici*).

The asexual life cycle of *B. graminis*, from the beginning of the infection to the production of new spores, completes within a week. The haploid asexual fungal spores, called conidia, start germination within a few hours after contact with a plant leaf. The appressorial germ tube penetrates the cell wall of the leaf epidermal cells directly and grows into the living plant cell forming a feeding structure called a haustorium. The establishment of biotrophy occurs within the first 24 hours after leaf spore inoculation. In the following days, epiphytically growing hyphae develop many secondary haustoria in neighboring epidermal cells next to the initial infection site. After three days, the fungal colony is macroscopically visible. In the following days, abundant spores are formed by the mycelium, which completes the life cycle [5]. In controlled infection assays with defined spore titters, the severity of the infection and the size of the infected area are commonly the scoring parameters in disease rating to estimate host susceptibility [6].

Cultivated barley (*Hordeum vulgare* spp. *vulgare*), a member of the *Triticeae* tribe of grasses, is among the most favored crops worldwide. Besides, barley is a famous genetic model for the very closely related but more complex wheat genome.

With the significant progress made on the sequencing of several cereal genomes, Genome-Wide Association Studies (GWAS) to identify resistance traits became possible. However, a bottleneck for successful genotype-phenotype associations is the high-throughput monitoring of disease symptom development as a measure of host plant susceptibility. Disease resistance traits range from partial, or quantitative, to complete, or qualitative. It has been shown in many cases that quantitative disease resistance is more durable on the field and, therefore, of high potential value to the breeders [7]. However, the quantitative resistance is usually a polygenic trait, which is based on the joined effect of many genes, where each of them contributes quantitatively to the level of plant defense [8]. The identification of genes with small to moderate resistance effects requires exact and reproducible quantification of infection as a prerequisite for genetic fine mapping and gene isolation.

The choice of high-throughput phenotyping technologies for disease resistance has rapidly increased over the last years. There are methods based on measuring the enzymatic activity of the infected tissue [9], on chlorophyll fluorescence [10], or on quantitative PCR of fungal genes [11], but more commonly optical sensors and computer vision approaches are used [12]. Hyperspectral imaging is using the information about the reflectance of the tissues in a wide range of wavelengths and may visualize the disease symptoms in relatively early stages [13, 14]. Multispectral imaging is done with only a few but usually highly informative wavelengths, thus significantly reducing the cost of equipment and the amount of raw data. However, the most common type of optical sensors is using the visible and near-visible spectrum. These sensors are either with integral wide-band filter matrices for limiting the sensitivity on the pixel level to specific wavelengths (e.g., RGB cameras) or without wavelength discrimination (grayscale cameras) but often with external filters and/or illumination sources with a discrete wavelength band.

Although the phenomics platforms are typically built on highly customized hardware, some implementations are using standard hardware components from the consumer market. This approach allows the building of very cost-efficient and versatile systems but with certain limitations. An example of such a system represents the PhenoBox [15], which is based on a consumer mirror-reflex camera for phenotyping in controlled conditions. The system allows phenotyping of biotic and abiotic stress of small plants and plant organs at a very moderate cost. However, the inbuilt filters of the used camera type allow imaging only in the visible range of light.

Designs based on multiple single-board computers (e.g. Raspberry Pi and Arduino) provide another exciting low-cost alternative to the large robotics platforms. An example of such a system is the low-cost SeedGerm platform [16], which is using multiple Raspberry Pi computers and implements a single-axis camera movement for seed imaging and germination phenotyping. Nevertheless, typically the low-cost devices are usually missing robotics components for sample handling that renders them less appropriate for high-throughput and 24/7 applications.

The development of image analysis methods is very dynamic and contributes to increasing the expectations for more complex phenotypes. Currently, two primary approaches are commonly used to analyze image data. The first one is using custom handcrafted features, and the other one is based on Artificial Neural Networks (ANN). The first approach typically requires a significant manual input in the form of selecting and implementing informative features, but efficient models can be built with a relatively low amount of training data, which makes this approach attractive in some instances [17, 18].

The current state of the art machine learning methods is based on ANN and most recently deep learning (DL) architectures [19–22]. However, early DL architectures are less appropriate for semantic segmentation (pixel-based classification) because of the missing spatial information, which is an essential feature for conventional image recognition networks such as convolutional neuronal networks [23]. Therefore, edge detection, clustering, or thresholding-based segmentation solutions are still frequently used for pixel classification [24]. Another drawback of the DL is the typically very high demand for annotated training samples and high hardware requirements. New Convolutional Neural Network (CNN) architectures like Mask Regional-CNN (Mask R-CNN) [25] or U-Nets CNN [26] promise to solve many of the limitations of the DL and are already used for semantic segmentation of plant diseases [27, 28].

This work is aimed at establishing a high-throughput, automated phenotyping platform for precise and reproducible quantification of leaf disease of cereals, with a focus on powdery mildews and rusts.

## 2. Materials and Methods

### 2.1. Experimental Design

**Figure 1:**
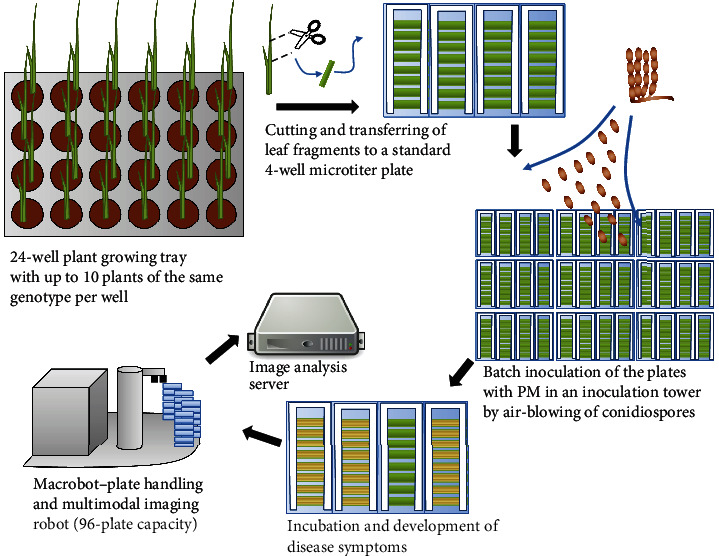
Overview of the phenotyping pipeline. The plants are grown in 24-well trays in a greenhouse. At the appropriate stage, leaf fragments are harvested and mounted on standard 4-well microtiter plates, filled with 1% water agar for keeping the humidity, and inoculated by air-blowing of powdery mildew spores in an inoculation tower. After incubation of 5-7 days, the disease symptoms become visible. The plates with the infected leaves are loaded into the Macrobot system for automated imaging. The acquired images are transferred to the image analysis server for quantification of the disease symptoms.

The Macrophenomics pipeline consists of hardware and software components. A specialized robotic system (Macrobot) implements the image acquisition part of the Macrophenomics pipeline. The Macrobot autonomously acquires images of detached leaf segments mounted on standard size microtiter plates (MTPs) (Figure 1).

Typically, the wheat and barley plants are grown in 24-well trays in a greenhouse. The samples are taken at the 2-leaf stage from the middle part of the second leaf. The leaf fragments are mounted on standard 4-well MTPs with 1% water agar (Phyto agar, Duchefa, Haarlem, the Netherlands) supplemented with 20 mg L^−1^ benzimidazole as a leaf senescence inhibitor. For achieving regular inoculation of all leaves, the plates without lids are placed in a rotating table inside an inoculation tower and are inoculated by blowing in conidiospores from sporulating material. Inoculated plates are incubated in environmentally controlled plant growth chambers (20°C, 60% RH constant; 16 h light, 15 *μ*E m^−2^ s^−1^) for 6 days until the disease symptoms are visible. The infected plates are loaded into the Macrobot system for automated imaging. The acquired images are transferred to the image analysis server for quantification of the disease symptoms.

### 2.2. Hardware

In the original version, the Macrobot employs a 14-bit monochrome camera (Thorlabs 8050M-GE-TE) at a resolution of 3296 × 2472px. A high-end lens (CoastalOpt UV-VIS-IR 60 mm 1 : 4 Apo Macro) with apochromatic correction in the range from 310 to 1100 nm wavelength ensures that images using different illumination setups are precisely registered and focused. The illumination is realized using small-bandwidth isotropic LED light sources (Metaphase Exolight-ISO-14-XXX-U) with 365 nm (UV), 470 nm (blue), 530 nm (green), and 625 nm (red) peak wavelengths.

The robotic part of the Macrobot is built of off-the-shelf OEM components developed for laboratory use that facilitate the maintenance and allow quick replacement of defective components. Specifically, a PlateCrane EX Microplate Handler (Hudson Robotics, Inc., NJ, USA) in conjunction with an IGUS precision linear stage (igus GmbH, Germany) is used. The plate crane and linear stage can be accurately positioned with high repetition accuracy using the vendors' software API. The positions of the plate crane and linear stage for the different stages of plate handling are determined at the initial system setup and have to be manually readjusted if the system was moved or rearranged in some form. Positional information is stored in parameter files and reloaded in the Fraunhofer IFF custom implemented system software. This also applies to the imaging settings of the Thorlabs camera. The imaging process is controlled by the system software using its custom scripting engine, which allows a controlled complex sequence of steps to unfold in order to ensure a repeatable and high-throughput measurement on the supplied sample set. Imaging procedure parameters can be adjusted by the user and are stored as profiles to ensure repeatable imaging.

For each plate, monochrome images in all illumination wavelengths are acquired separately and stored in 16-bit TIFF image files. An RGB image is generated by combining the images of the red, green, and blue LED channels (Supplemental Figure S1). The UV channel is used to facilitate the extraction of the region of interest (ROI), where the leaves are located. Video sequences showing the Macrobot in action can be seen in [29].

**Figure 2:**
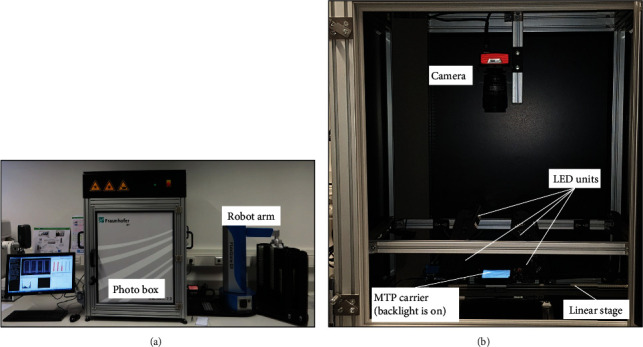
Macrobot 2.0 with improved technical design, bilateral illumination, and background light: (a) outside view and (b) inside view of the photo box.

An improved version of Macrobot was introduced on a later stage and designated as Macrobot 2.0 (Figure 2). The illumination system was upgraded by doubling the LED units allowing bilateral illumination of the objects. A background illumination system based on electroluminescence foil was mounted on the MTP carrier to simplify the separation between the foreground and background, thus improving the leaf segmentation. The image acquisition and hardware controlling software was upgraded to a 64-bit version for optimal system memory utilization. The entire technical layout was improved with respect to the gained experience with the first version of the Macrobot. Since the image acquisition components remain unchanged, data generated by Macrobot 2.0 is fully comparable to data acquired by Macrobot 1.0, as far as a comparable hardware setup is used (e.g., one-sided illumination). The data presented in this article was acquired by the original Macrobot hardware configuration.

### 2.3. Software

The image analysis software was implemented in Python 3.8 under Microsoft Windows 10 with extensive use of the NumPy (v. 1.12.1) [30], opencv-python (v. 2.4.13), scikit-learn (v. 0.17.1) [31], and scikit-image (v. 0.13.0) [30] open-source libraries. The source code is available at [32].

### 2.4. Model Evaluation

Each model was validated by calculating the accuracy, recall, and precision of the model to test the prediction performance for each class. The overall accuracy is calculated by the number of correctly predicted observations divided by the total number of observations:

(1) $$\begin{array}{c} \text{Accuracy}=\frac{\text{TP}+\text{TN}}{\text{TP}+\text{FP}+\text{FN}+\text{TN}}. \end{array}$$

Precision is a measure of the false-positive rate. It can be calculated by dividing the true positive observations by the total predicted positive observations:

(2) $$\begin{array}{c} \text{Precision}=\frac{\text{TP}}{\text{TP}+\text{FP}}. \end{array}$$

Recall measures the sensitivity of the predicted positive observations:

(3) $$\begin{array}{c} \text{Recall}=\frac{\text{TP}}{\text{TP}+\text{FN}}. \end{array}$$

### 2.5. Wheat Genotype Collection

A collection of 188 elite lines and 202 genetic resource lines was obtained from the federal ex situ collection of the Gene Bank of IPK Gatersleben. The complete list of the genotypes is given in Supplemental Table S6.

### 2.6. Best Linear Unbiased Estimations (BLUEs) of Genotype Response against Fungal Infections

The phenotypic results of the tested genotype collection are represented as BLUEs obtained by fitting the data to the following linear mixed model:

(4) $$\begin{array}{c} y=1_{n}\mu +X_{G}g+Z_{R}r+e, \end{array}$$

where *y* is the vector containing the *n* original raw fungal infection data points, 1_*n*_ indicates an *n*-size vector of only 1 s, *μ* stands for the fixed intercept term, *g* is a vector of the fixed genotypic effects of the material being tested, and *r* represents a vector that contains all random factors beyond residual variation: experiment, treatment, the interaction between experiments and genotypes, and the interaction between genotypes and treatments, while *e* indicates a vector of random residual variation. *X*_*G*_ and *Z*_*R*_ are design matrices that assign *g* and *r* to the corresponding values contained within *y*. Mixed model equations were computed using the lme4 package [33] implemented in R Environment ver. 3.4.0 [34].

### 2.7. Plant and Fungal Material

Wheat and barley plants from different cultivars and landraces were grown in 24-pot trays (31 × 53cm) in a greenhouse at 20°C constant and 16 h light period in a soil substrate. The first or the second leaves were harvested at 7 days and 13-14 days, respectively, after sowing. The leaf segments were mounted on 20 mg L^−1^ benzimidazole-supplemented and 1% water agar plates and inoculated with the corresponding pathogen at approximately 10 spores/mm^2^. As pathogens, the Swiss wheat powdery mildew field isolate FAL 92315 and the Swiss barley powdery mildew field isolate CH4.8 were used, respectively. The image acquisition was performed seven days after inoculation (dai).

#### 2.7.1. Wheat Genotype Collection

A collection of 187 elite lines and 201 plant genetic resource lines was obtained from the federal ex situ collection of the Gene Bank of IPK Gatersleben. The complete list of the genotypes is given in Supplemental Table S6.

### 2.8. Quantitative PCR

Quantitative real-time PCR was performed in a volume of 5 mL QuantiTect Probe PCR Kit (Qiagen GmbH, Hilden, Germany) and an ABI 7900HT fast real-time PCR system (Thermo Fisher Scientific Inc., Waltham, MA, USA). Forty cycles (15 sec, 94°C; 30 sec, 56°C; 30 sec, 72°C, preceded by standard denaturation steps at 94°C for 2 min) were conducted. Data were analyzed by the standard curve method using the SDS 2.2.1 software (Thermo Fisher Scientific Inc., Waltham, MA, USA). A standard curve dilution series was included for each gene, as fivefold dilutions and three technical replicates per DNA sample. The detected quantity of the fungal gene GTFI (beta-1,3-glucanosyltransferase, GenBank: EU646133.1) was normalized to the quantity of the barley UBC gene (ubiquitin-conjugating enzyme, GenBank: AY220735.1) and used as a proxy for fungal biomass.

The used primers and probes are as follows: for the powdery mildew GTFI gene—BgGTF1_F (5′TTGGCCAAACAACTCAACTC3′), BgGTF1_R (AGCAGACCAAGACACACCAG), and BgGTF1_PR (fluorescent TaqMan probe, FAM-5′CTCCCAGCAACACTCCAGCT3′-BHQ1), and for the barley UBC gene—HvUBC_F (5′ACTCCGAAGCAGCCAGAATG3′), HvUBC_R (5′GATCAAGCACAGGGACACAAC3′), and HvUBC_PR (fluorescent TaqMan probe Yakima Yellow-5′GAGAACAAGCGCGAGTACAACCGCAAGGTG3′-BHQ1).

## 3. Results

### 3.1. Frame and Leaf Segmentation

**Figure 3:**
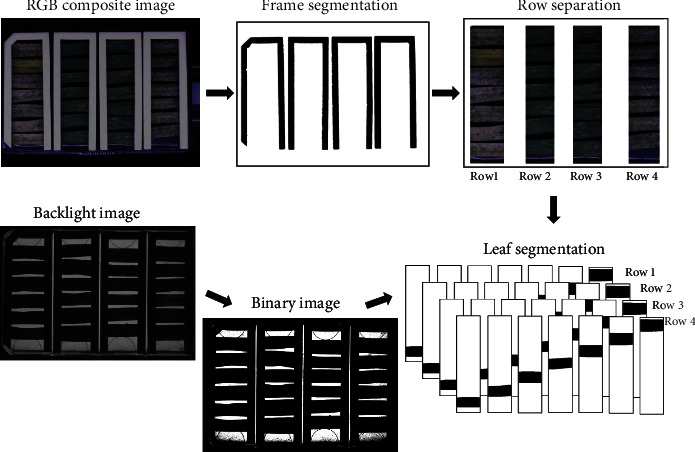
Frame and leaf image segmentation processing chain. In the first step, the white leaf-holding frames are used to define the regions of interest (ROIs) that contain the leaf fragments. Next, the plate image is split on four ROIs, each one containing a row of leaf samples that typically belong to a single genotype but to a different individual plant (e.g., each leaf fragment in a row is a technical replicate). In parallel, the back-illuminated image of the plate is used to separate the leaves of each other. The final leaf segmentation is done by combining the row and leaf ROIs.

To define the area where the leaf segments are located on the plates, the C-shaped white frames that hold the leaves were segmented and extracted. Optimal results were achieved by applying Otsu's thresholding [35] on the UV channel, followed by dilation with an 8 × 8 kernel to obtain a binary image (Figure 3). The Moore-Neighbor [36] tracing was used to extract the contours of the binary image and filter the frames by size and position.

Each leaf segment was extracted to a separate region of interest (ROI). The best segmentation results were obtained by Otsu's binarization method on the backlight image, followed by the Moore-Neighbor contour finding algorithm and object size selection. Otsu's method also gets along with the particular challenge of interrupted leaf contours caused by necrosis or fungal infections.

### 3.2. Machine Learning Approach

The application of machine learning approaches gives the advantage of using a data-driven analysis rather than hypothesis-driven statistics. In this way, complex statistical modeling assumptions can be reduced, offering possibly important data features from which machine learning tools can derive desired classification outcomes in the manner of teaching. Therefore, several machine learning methods were implemented and evaluated for their accuracy and performance in the quantification of the PM disease symptoms.

#### 3.2.1. Training Data

Training data was collected by manual labeling of background, infected, and leaf necrosis areas. The single labeled pixels were extracted and assigned to these three classes. For avoiding a class imbalance, the number of training samples per class was adjusted to the lowest number of pixels per class, which was 5000. The dataset was split: 70% for training and 30% for validation.

#### 3.2.2. Feature Extraction and Classification

**Figure 4:**
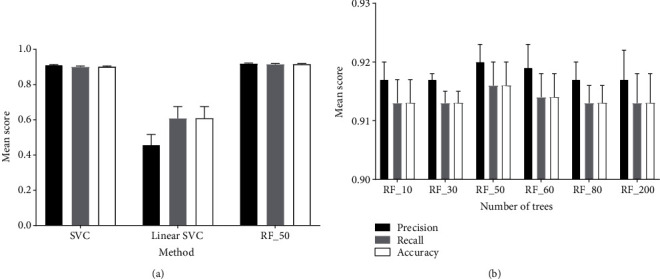
(a) Evaluation of different classifiers on HSV_H_channel (5000 pixels/class, *n* = 10, errorbars = SD). (b) Evaluation of the random forest classifier with different numbers of trees on HSV_H_channel (5000 pixels per class, *n* = 10).

We have compared three conventional classifiers: C-Support Vector Classification [37], Linear Support Vector Classification [38], and random forest [39]. We found the random forest to be performing significantly better than the Linear Support Vector Classification and slightly better compared to the C-Support Vector Classification (Figure 4(a), Supplemental Table S2). The training time with the random forest classifier was about ten times faster than that with the C-Support Vector Classification. Therefore, we end up using the random forest classifier for further experiments.

To find the optimal number of trees for the random forest classifier, we tested six different values ranging from 10 to 200 trees, which lead to an optimum of 50 trees (Figure 4(b), Supplemental Table S3).

**Figure 5:**
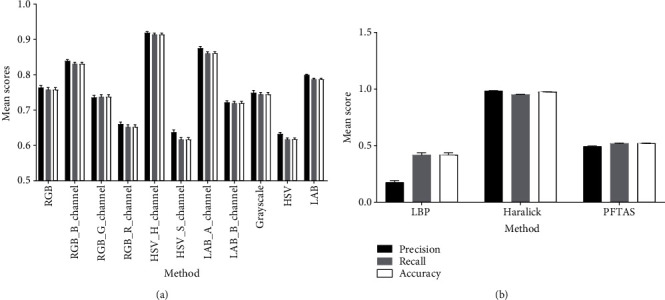
(a) Evaluation of different color pixel classification methods (5000 pixels/class, *n* = 10, errorbars = SD). Random forest classifier (nr_trees = 50). (b) Evaluation of texture features (5000 pixels/class, *n* = 10, errorbars = SD). Random forest classifier (nr_trees = 50).

A random forest classifier has been trained by using RGB, Lab, and HSV as multiple- and single-color channels (Figure 5(a), Supplemental Table S4).

Texture spatial features such as local binary pattern [40], Haralick [41], and Parameter-Free Threshold Adjacency Statistics (PFTAS) [42] were also tested for improving the performance of the classifier (Figure 5(b), Supplemental Table S5).

Four models reached an overall accuracy above 0.80: the blue channel of the RGB color space, the hue channel of the HSV color space, the *a* channel of the Lab color space, and the Haralick texture features. Those models were tested further in the validation experiment.

### 3.3. Segmentation Approach

**Figure 6:**
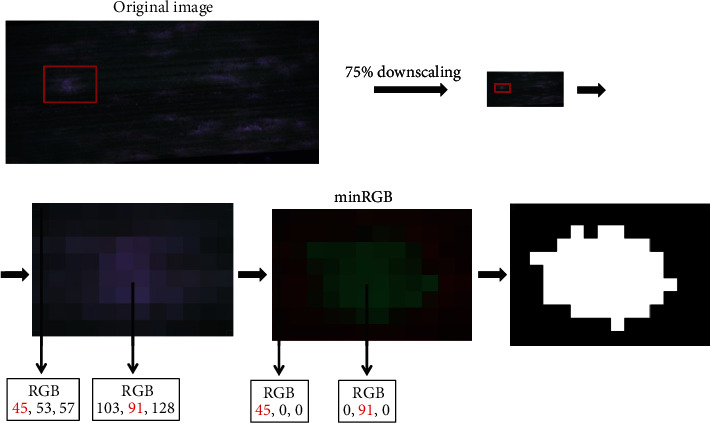
Minimal RGB value approach for segmentation.

In addition to the machine learning approach, we have tested several segmentation methods: edge detection, superpixel segmentation, watershed transformation, region growing methods, thresholding, and minimum and maximum RGB (data not shown). The most efficient segmentation was achieved by the relatively simple method of minimum RGB (minRGB) (Figure 6). The algorithm takes the single values for each RGB channel, determines the minimum number of each channel, and stores the value. The other two channels are set to the value 0. This simple filter allowed reliable differentiation of the disease symptoms from the background by simultaneous reduction of the analysis artifacts and hardware workload.

### 3.4. Validation Experiment

The Macrophenomics module is aimed at providing a precise and reproducible evaluation of the experimental results and at the same time at releasing the human personnel from a routine and laborious task. To estimate the performance of the different approaches and computer models on independent material, we have carried out a validation experiment, where six domain experts were asked to do a manual disease rating. Combining the scores given by all experts formed a robust mean value, which was used to validate the computer prediction results. The validation set included a partially very difficult to score material with a lot of leaf senescence and necrosis.

**Figure 7:**
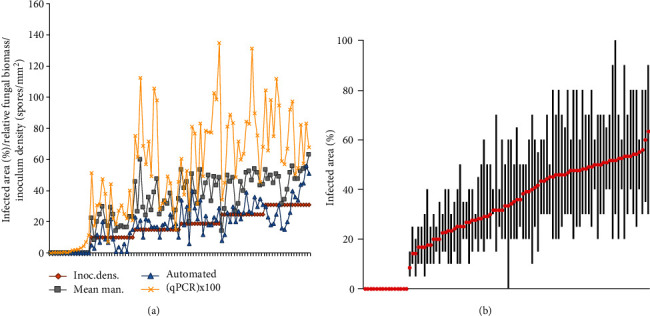
(a) Plots of the infection area determined automatically (blue triangles) and mean manual values (“Mean man.,” green rectangles), together with the fungal biomass measured by qPCR (normalized relative transcript levels multiplied by 100 for better visibility, purple crosses) and inoculation density (spores per mm^2^, red rectangle, sorted ascending). On the *x*-axis are the ordered samples, and on the *y*-axis are the infection area (% of the leaf surface), relative fungal biomass (relative units), and inoculation density (spores/mm^2^). (b) The minimal and maximal visual infection scores (black bars) and the means (red dots) estimated by the domain experts. The graphs show the discrepancy of visual scoring of the involved persons. On the *x*-axis are the ordered samples sorted by the mean, and on the *y*-axis is the infection area (% of the leaf surface).

In parallel to the visual methods, two other types of measurements were included for comparison: quantification of the total fungal biomass using quantitative real-time PCR (qPCR) of fungal DNA and inoculum density as the number of applied fungal spores per mm^2^ of leaf surface (Figure 7(a), Supplemental Table S1).

#### 3.4.1. Performance of the Machine Learning Approach

**Figure 8:**
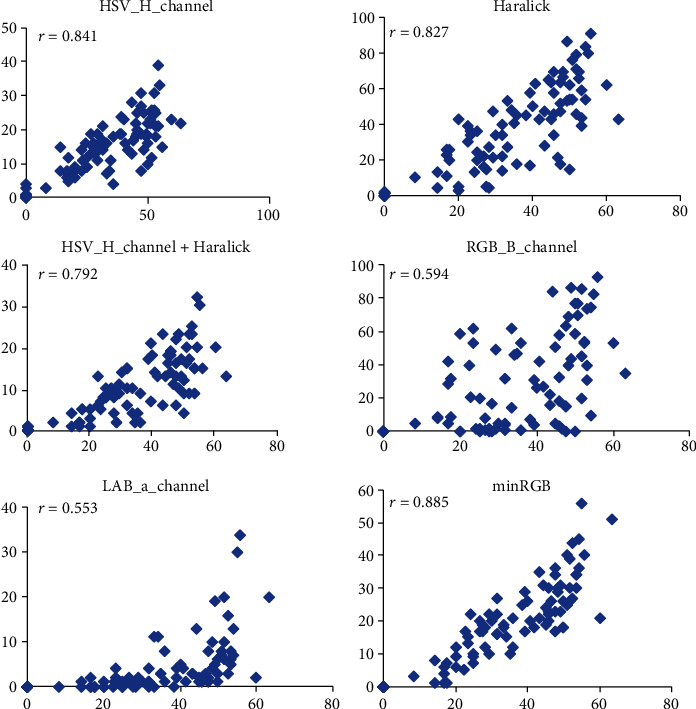
Scatter plots and Pearson's coefficients of correlations (*r*) for the different machine learning models plus the minRGB-based segmentation (*y*-axis) versus the mean visual scores given by six experts (*x*-axis). PM infected detached barley leaves, 6 dai. The number of samples (*n* = 108).

All machine learning models with accuracy above 0.8 (Figures 4 and 5) plus the minRGB segmentation algorithm were tested on the validation experiment data by comparing the mean visual scores and the predictions of the corresponding models (Figure 8).

The best correlation to the mean visual data (0.885) was achieved by the minRGB method, confirming once more its efficiency for image segmentation of the particular material.

#### 3.4.2. Performance of the Segmentation Approach

**Figure 9:**
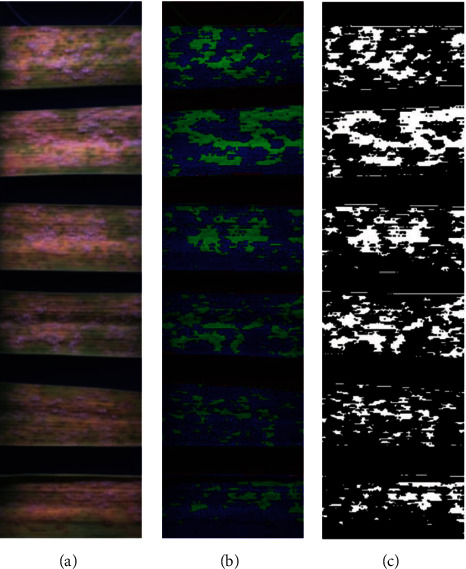
An example of a minRGB filter-based detection of the disease area on PM-infected detached barley leaves 6 dai. (a–c) RGB composite image, minRGB filter results, and final prediction after thresholding.

Although the leaf material of the validation experiment was often covered by large necrotic and/or chlorotic areas, which may complicate the disease recognition even for domain experts, the minRGB-based prediction was very accurate (Figure 9).

**Figure 10:**
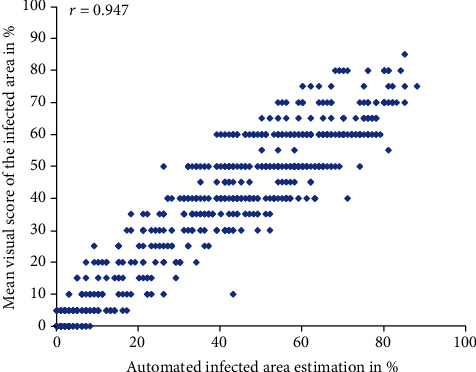
Scatter plot and Pearson's coefficients of correlation (*r*) between the visual score given by an expert (*y*-axis) and minRGB-based automated scores for the PM-infected area (*x*-axis), 6 days after infection. Number of samples (*n* = 660) and number of testers (*p* = 1). The manual scores were given in 5% steps.

The minRGB-based algorithm was also tested in a larger experiment with wheat, showing an even higher level of accuracy (Figure 10).

The better results for the wheat material might be explained with the lower frequency of appearance of problematic artifacts such as necrosis and senescence in this particular material.

The run time per sample and per dataset was reduced up to 10-fold by using the minRGB approach in comparison to the per-pixel classification methods. With the particular hardware configuration, the image analysis time was up to 3-fold shorter than the time required for image acquisition, thus allowing the implementation of image analysis in real time.

### 3.5. Application of the Macrophenomics Platform for Evaluation of Quantitative Disease Resistance of Genetically Diverse Plant Material

The disease phenotype may strongly depend on the genotype of the host plant. Therefore, we have tested a collection of 388 genetically diverse wheat genotypes for quantitative disease resistance against wheat powdery mildew. The collection consists of 187 elite breeding lines, released post-Second World War, and 201 plant genetic resources, mostly historically collected landraces. The plants were grown to the two-leaf stage (13 days), and the second leaf was cut and inoculated with the wheat powdery mildew isolate FAL 92315. The disease symptoms were scored after 6 days of incubation at 20°C, 60% RH, and 16 h light period. Each genotype was tested in two biological repetitions, with six individual plants per genotype.

**Figure 11:**
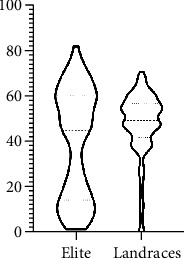
Distribution of the BLUEs for powdery mildew disease resistance in 5250 individual plants from two biological replicates. The elite line pool consists of 187 genotypes; the plant genetic resources (landraces) consist of 201 genotypes. The higher BLUEs indicate higher susceptibility to the pathogen. The elite lines show significant enrichment of disease-resistant genotypes.

The result showed a very different distribution of the levels of quantitative disease resistance within the two groups, with a significant enrichment of resistance genotypes in the elite lines (Figure 11).

## 4. Discussion

A massive body of data on powdery mildew disease resistance of different plant genotypes was collected over the years by the breeders and researchers. Although a precious resource, the majority of this data is hardly reproducible because it is collected mostly in uncontrolled field conditions, with an unknown mix of pathogen isolates, and visually scored by many different persons. To overcome these problems and to establish a platform for precise phenotyping under controlled conditions, we have developed a high-throughput platform for phenotyping of cereal leaf diseases. The system is based on the well-established detached leaf assay [43–45], which allows a very high level of controlling the environment and pathogen pressure. The method is also very well adapted for phenotyping via optical sensors since the leaves can be mounted in special containers for acquiring images in fully controlled conditions.

In our system, we have selected a monochrome CCD sensor to avoid some of the inbuilt problems of the RGB cameras (e.g., pixel value interpolation and lowered quantum efficiency). Instead of using filters for specific wavelengths, we decided to use narrow-bandwidth isotropic LED light sources, thus avoiding the use of motorized filter magazines and losing quantum efficiency. The nature of the samples (nonmoving fixed objects) allows the acquisition of several images per object and the combination of the data without complicated merging methods. The leaf samples are fixed in standardized containers (microtiter plates), which greatly simplify the hardware design allowing the use of commercially available components such as the plate crane. The white plastic frames that keep the leaves fixed in the plates are at the same time used to define the area of interests, where the leaves are located.

The central hardware part of the system is the Macrobot. It is equipped with custom imaging system software developed by Fraunhofer IFF (Magdeburg, Germany). Several software modules control all actors and sensors in the system providing services to a service manager. The flow control for the imaging process is achieved by script programming, which enables a change in the imaging process without reimplementing the different software modules and makes extensions to the system secure and efficient. System modules providing a graphical user interface are organized in a reconfigurable user interface, which can be arranged to the needs of the system user without reimplementation. The imaging system generates a structured dataset for the subsequent image analysis.

We have tested several machine learning and segmentation approaches to find the most efficient algorithm for disease quantification. The most informative features for the machine learning approach were the *H*, *B*, and *a* channels of the HSV, RGB, and Lab color spaces, respectively. Among the tested texture features, Haralick was the by far most informative. A combined pixel classification based on color and texture features was tested as well but without significant improvement compared to the single features. Of the three different classifiers that we have evaluated, the random forest (RF) performed slightly better than the Support Vector Classifier (SCV) and much better than the Linear SVC. We have tested also RF with a different number of trees, and we found the number of 50 trees to be optimal.

Astonishingly, among all tested segmentation approaches, the most accurate and efficient technique was the simple method of minimum RGB (minRGB). This filter was able to detect the infected leaf area reliably and to reduce the signal from disease-unrelated necrotic brown spots, which were of a particular problem in nearly all other approaches. Besides, the hardware workload and the calculation time for computing the minRGB filter were significantly lower than those of any other method. Finally, the minRGB was the segmentation method of choice, which was implemented into the image analysis pipeline.

A take-home message from this result can be that the use of sophisticated image analysis methods should not become a goal *per se*. In several cases, more straightforward methods may provide comparable results at a much lower cost.

We have validated the prediction results by three other direct and indirect quantification methods: a visual scoring as the mean value of the scores of six different domain experts, quantitative PCR (qPCR), and inoculum density as the number of spores per square millimeter of the leaf surface.

Although the genomic qPCR provides a nearly direct estimation of the total fungal biomass, it is a complicated method, which is influenced by many factors, such as genomic DNA isolation and quality, primer design, PCR efficiency, and detection sensitivity. Also, the measured quantity depends on both visible (on the leaf surface) and invisible (too small or internal) fungal structures and is therefore not necessarily in perfect correlation with the visible disease symptoms. The inoculum density is instead an indirect parameter, which gives the infection pressure and the potential for the formation of fungal colonies. However, the formation of the final fungal biomass depends on several other biotic and abiotic factors, such as spore fitness and aggressivity, plant response, and support of the fungal growth and temperature and humidity. The mean scoring value of several persons provides a very robust parameter, and therefore, it was the method of choice for calibration of the automatic prediction.

We have applied the Macrobot platform for the evaluation of an extensive, diverse collection of genotypes. The collection contains modern breeding material (elite lines) and a historical collection of landraces and semiwild genotypes. Widespread opinion among the nonexperts and some experts is that the wild species and landraces are typically more resistant to biotic and abiotic stress than the modern high-yielding varieties. Interestingly, our observation demonstrates that the elite lines showed a substantial enrichment of resistant genotypes compared to the landraces. This observation reflects the long-term efforts of the breeders for introducing disease resistance alleles in the elite material. These results prove the capability of the Macrobot system to deliver high-quality data for very diverse plant material. The obtained phenotypic data can be used directly for breeding purposes or GWAS approaches.

In this work, we demonstrate that our Macrophenomics platform can provide reliable and reproducible data in an excellent correlation with the classical scoring methods, and it can even outperform the scores of individual experts by the accuracy of infection area estimation. The platform is also fully open for adaptation to diseases other than powdery mildew leaf diseases such as different spot, blight, and rust diseases caused by several fungal, viral, and bacterial pathogens, such as yellow and brown rusts (*Puccinia* sp.), septoria leaf blotch (*Zymoseptoria tritici*), spot blotch (*Bipolaris sorokiniana*), bacterial leaf blight (*Pseudomonas syringae*), bacterial leaf streak and black chaff (*Xanthomonas translucens*), and barley yellow dwarf virus. However, an important limitation is that the tested objects must fit into a standard MTP container (app. 12 × 8 × 1cm), which includes samples like detached leaves, seeds, stem and root fragments, cereal spikes, and small whole plants.

A FAIR principle [46] compliant data management is currently under development. The pipeline follows the basic schema of the BRIDGE Visual Analytics Web Tool for Barley Genebank Genomics [47]. It will allow visualization and export of the phenotypic and genotypic data, online GWAS, and several other features of interest to the scientist and plant breeders.

## Data Availability

Image data used for validation of the Macrobot algorithm is available at [48]. The image analysis software is available at [32].

## Supplementary Materials

Supplementary Materials Supplemental Figure S1: schematic drawing of the image acquisition hardware of the Macrophenomics module Macrobot 2.0 (top and side views). Supplemental Table S1: validation experiment, all data (Excel sheet). Supplemental Table S2: values in Figure 4(a)—evaluation of different classifiers on HSV_H_channel (5000 pixels per class, *n* = 10). Supplemental Table S3: values in Figure 4(b)—evaluation of the random forest classifier with a different number of trees on HSV_H_channel (5000 pixels per class, *n* = 10). Supplemental Table S4: values in Figure 5(a)—evaluation of different color pixel classification methods (*n* = 10). Supplemental Table S5: values in Figure 5(b)—evaluation of texture features (*n* = 10). Supplemental Table S6: list of wheat genotypes (Excel sheet).
