# Supplementary material for: “Macrobot”: An Automated Segmentation-Based System for Powdery Mildew Disease Quantification
Source: Plant Phenomics. 2020 Nov 5;2020:5839856. doi: 10.34133/2020/5839856 (PMC7706317; doi:10.34133/2020/5839856)
Supplement: Supplementary Materials — Supplemental Figure S1: schematic drawing of the image acquisition hardware of the Macrophenomics module Macrobot 2.0 (top and side views). Supplemental Table S1: validation experiment, all data (Excel sheet). Supplemental Table S2: values in Figure 4(a)—evaluation of different classifiers on HSV_H_channel (5000 pixels per class, n = 10). Supplemental Table S3: values in Figure 4(b)—evaluation of the random forest classifier with a different number of trees on HSV_H_channel (5000 pixels per class, n = 10). Supplemental Table S4: values in Figure 5(a)—evaluation of different color pixel classification methods (n = 10). Supplemental Table S5: values in Figure 5(b)—evaluation of texture features (n = 10). Supplemental Table S6: list of wheat genotypes (Excel sheet). [file 5839856.f1.zip › Supplemental figures and tables.docx]

# Supplemental figures and tables

**Supplemental figure S1.** Schematic drawing of the image acquisition hardware of the Macrophenomics module Macrobot 2.0 (top and side views).

**Supplemental Table S1**: Validation experiment, all data (Excel sheet)

**Supplemental Table S2.** Values to **Figure 4A** - Evaluation of different classifiers on HSV_H_channel (5000 pixels per class, n=10).

| Method | Precision | SD | Recall | SD | Accuracy | SD |
| --- | --- | --- | --- | --- | --- | --- |
| SVC | 0.912 | 0.003 | 0.902 | 0.004 | 0.902 | 0.004 |
| LinearSVC | 0.458 | 0.059 | 0.609 | 0.066 | 0.609 | 0.066 |
| RF_50 | 0.920 | 0.003 | 0.916 | 0.004 | 0.916 | 0.004 |

**Supplemental Table S3.** Values to **Figure 4B** - Evaluation of Random forest classifier with a different number of trees on HSV_H_channel (5000 pixels per class, n=10).

| Number of trees | | Precision | | SD | Recall | | | SD | Accuracy | | | SD |
| --- | --- | --- | --- | --- | --- | --- | --- | --- | --- | --- | --- | --- |
| 10 | | 0.917 | | 0.003 | 0.913 | | | 0.004 | 0.913 | | | 0.004 |
| 30 | | 0.917 | | 0.001 | 0.913 | | | 0.002 | 0.913 | | | 0.002 |
| 50 | 0.920 | | 0.003 | | | 0.916 | 0.004 | | | 0.916 | 0.004 | |
| 60 | 0.919 | | 0.004 | | | 0.914 | 0.004 | | | 0.914 | 0.004 | |
| 80 | 0.917 | | 0.003 | | | 0.913 | 0.003 | | | 0.913 | 0.003 | |
| 200 | 0.917 | | 0.005 | | | 0.913 | 0.005 | | | 0.913 | 0.005 | |

**Supplemental Table S4**. Values to **Figure 5A** - Evaluation of different color pixel classification method (n=10)

| Method | Precision | SD | Recall | SD | Accuracy | SD |
| --- | --- | --- | --- | --- | --- | --- |
| RGB | 0.7632 | 0.0062 | 0.7577 | 0.0059 | 0.7577 | 0.0059 |
| RGB_B_channel | 0.8390 | 0.0041 | 0.8308 | 0.0046 | 0.8308 | 0.0046 |
| RGB_G_channel | 0.7355 | 0.0063 | 0.7374 | 0.0063 | 0.7374 | 0.0063 |
| RGB_R_channel | 0.6601 | 0.0058 | 0.6521 | 0.0065 | 0.6521 | 0.0065 |
| HSV_H_channel | 0.9189 | 0.0034 | 0.9140 | 0.0038 | 0.9140 | 0.0038 |
| HSV_S_channel | 0.6371 | 0.0068 | 0.6167 | 0.0056 | 0.6167 | 0.0056 |
| LAB_A_channel | 0.8750 | 0.0053 | 0.8605 | 0.0047 | 0.8605 | 0.0047 |
| LAB_B_channel | 0.7214 | 0.0050 | 0.7195 | 0.0051 | 0.7195 | 0.0051 |
| Grayscale | 0.7487 | 0.0071 | 0.7446 | 0.0055 | 0.7446 | 0.0055 |
| HSV | 0.6324 | 0.0044 | 0.6172 | 0.0039 | 0.6172 | 0.0039 |
| LAB | 0.7994 | 0.0020 | 0.7871 | 0.0031 | 0.7871 | 0.0031 |

**Supplemental Table S5**. Values to **Figure 5B** - Evaluation of texture features (n=10)

| Method | Precision | SD | Recall | SD | Accuracy | SD |
| --- | --- | --- | --- | --- | --- | --- |
| LBP | 0.1758 | 0.0159 | 0.4189 | 0.0190 | 0.4189 | 0.0190 |
| Haralick | 0.9836 | 0.0030 | 0.9522 | 0.0017 | 0.9746 | 0.0014 |
| PFTAS | 0.4950 | 0.0027 | 0.5202 | 0.0027 | 0.5202 | 0.0027 |

**Supplemental Table S6.** List of wheat genotypes (Excel sheet)
